# Information accessibility, accounting manipulation, and sustainable development of digital enterprises: Based on double moderating effect model and panel PSM-DID method

**DOI:** 10.1371/journal.pone.0283843

**Published:** 2023-03-31

**Authors:** Shujuan Wu, Minmin Li, Jianhua Xiao, Jianhua Tang

**Affiliations:** Economy and Management College, Wuyi University, Jiangmen, China; University of Murcia: Universidad de Murcia, SPAIN

## Abstract

A theoretical mechanism was analyzed from the micro perspective of the enterprise to explore how information accessibility moderates the effect of accounting manipulation on the sustainable development of digital enterprises. Using data from 1200 listing digital enterprises in China and the DEA-Malmquist index method, the efficiency value of digital enterprises in 2007–2021 was estimated to represent the index of sustainable development of digital enterprises. The accounting manipulation was detected using the panel PSM-DID method based on the Administrative Measures for the Recognition of High-tech Enterprise’s policy. The information accessibility value was estimated based on the MDA method. Empirical studies were conducted using text analysis, the panel PSM-DID method, and the double moderating effect model. The results showed that: (1) Accounting manipulation had a negative impact on the sustainable development of "true" digital enterprises and the "fake" digital enterprises. (2) Information accessibility directly and positively enhanced the technological progress and scale efficiency of digital enterprises, and its moderating effect was heterogeneous, with a significant moderating effect on the "true" digital enterprises and a negative effect on the "fake" ones.

## Introduction

Facing a new downward pressure on the economy [1], it is imperative to accelerate the economic transformation and upgrading and foster new impetus for economic development by empowering of the digital economy (DE) [2]. Therefore, policies such as the 14th Five-Year Plan for Digital Economy Development of China intend to invest more into the weak fields of the DE to break through the bottlenecks of DE development and promote the integration of the DE with the real economy. Implementing preferential policies (PP) to support the growth of a young industry, including the digital industry, is both a deliberate strategy and a common practice [3, 4]. However, policy preference (PP) induces accounting manipulation (AM), which undoubtedly affects the allocation of policy resources, and impedes the sustainable development of DE [5].

The rapid development of DE has significantly altered public administration and business operations. As a type of general application technology, taking the Internet as media, digital technology has been widely applied in public management [6], enabling remote and instant sharing of information, improving the accessibility of information for the authorities and the public, and subverting the traditional public management mode. Therefore, constructing and developing E-government facilitate the implementation of preferencial policies [7], limit the scope of AM, and weaken the impact of AM on the sustainable development of digital enterprises. In addition, using Internet-based information technology in enterprises improves the asymmetry of supply and demand information, the matching level between supply and demand, and production factor efficiency, thereby optimizing factor allocation efficiency. In other words, information accessibility (IA), which is improved because of DE development, is very likely to mitigate the negative impact of AM and optimize the sustainable development of DE.

However, several variables, such as the innovation and application stage of informatization, and the integration of the DE with real economy, still disrupt the overall impact of IA on AM. Currently, the digital technology is transitioning from the "installation" phase to the "development" phase [8]. An empirical question is how IA impacts the AM’s effect on the sustainable development of digital enterprises. The existing studies on the AM were abundant, from the perspectives of input factors allocation [9], ownership heterogeneity [10], corporate social responsibility’s effect on earnings management [11, 12], and the AM’s impact on the effect of PP [13], but the findings were controversial. In most cases, it was agreed that AM was detrimental to the sustainable development [14, 15] due to the authorities and the public’s limited identification ability and the information asymmetry. With the increasing integration of the DE and real economy, digital technology has empowered enterprise management and public management [16, 17]. It has an impact on AM and its impact on the sustainable development of enterprises.

Based on the case of digital enterprises and the Administrative Measures for the Recognition of High-tech Enterprises Policy (hereinafter referred to as the "Administrative Measures" or "the PP"), by using the exogenous event that digital enterprises were recognized as high-tech enterprises, this paper demonstrated:

1. The heterogeneous performance of "true" and "fake" digital enterprises’ efficiency, who were without or with AM, after being recognized by high-tech enterprises, to determine the impact of AM on the sustainable development of digital enterprises.
2. The direct impact of IA on the sustainable development of digital enterprises and its moderating effect of IA on the AM’s impact on the sustainable development of digital enterprises.

## Theoretical analysis and hypothesis

### AM affects the PP’s effect on the sustainable development of digital enterprises

The AM influences the distribution of the original policy dividends between the "true" and "fake" high-tech enterprises and impacts the effect on the sustainable development of "true" and "fake" digital enterprises heterogeneously.

#### AM’s impact on the "fake" digital enterprises’ efficiency

The effect mechanism is mainly through the following mechanisms:

First, the AM transfers a part of the policy dividends from “true” to "fake" high-tech enterprises. The ambitious "fake" digital enterprises increase investment for higher profits, which increases R&D investment [18], the demand for R&D labor, and the production and sales volume. However, the innovation facility and capacity of "fake" high-tech digital enterprises are weaker than the "true," resulting in a weaker technological progress effect.

Second, AM modulates the signal effect of PP. Due to AM, "fake" enterprises obtain policy R&D funds, non-R&D-specificity credit funds and risk ventures as part of a growing industry [19]. Typically, "fake" enterprises are relatively smaller, and higher possibility and financing translate to an expansion of R&D and production scale, resulting in increased efficiency. However, as income is a key prerequisite for policy dividend granting, a rational choice is to expand revenue scale, rather than R&D activity [20, 21]. The latter is of higher risk and uncertainty [22]. Therefore, the possibility of scale efficiency optimization of the "fake" digital enterprises is higher than that of technological efficiency and technological progress.

Third, the cost of AM diverts R&D investment, which hinders the optimization of technological progress and technological efficiency. The degree of the siphoning effect depends on the management decisions of the "fake" digital enterprises [23]. If R&D funds are misapplied to offset the cost of AM, it will hinder the technological progress of enterprises.

In conclusion, the impact of AM on the sustainable development of "fake" digital enterprises is extensive [24] and the net effect, which is determined by the balance of positive and negative effects, can be positive, negative or non-liner [25]. Therefore, the AM is very likely to affect enterprise efficiency, thereby influencing the sustainable development of digital enterprises. Therefore, the following hypothesis is proposed:

Hypothesis 1 (H1): *Under the premise of other conditions remaining unchanged*, *the AM has a positively impacts the sustainable development of the "fake" digital enterprises*.

#### AM’s impact on the "true" digital enterprises’ efficiency

There are two sides to AM’s impact on the sustainable development of "true" digital enterprises. One is that, with a larger number of policy beneficiaries, the policy dividend per capita of "true" digital enterprises is lower than before AM. The reduction of policy dividend weakens its original incentive effect on enterprises’ self-own R&D funds and the signal effect on R&D talents and financing, which has a negative impact on scale efficiency and technological progress. Along with the intense competition from "fake" enterprises [26], scale efficiency and technological progress of "true" digital enterprises have become negative. In addition, policy dividends’ multiplier effect and signal effect are conducive to maximizing efficiency. Therefore, the net efficiency effect of the AM on "true" digital enterprises’ sustainable development is decided by the counteractions of the positive and negative effects. Therefore, the following hypothesis is proposed:

Hypothesis 2 (H2): *Under the premise that other conditions remain unchanged*, *the AM has a negative effect on the sustainable development of the "true" digital enterprises*.

### The IA’s role in the AM’s impact on the sustainable development of digital enterprises

The impact of the IA in the AM’s effect on the sustainable development of PP is shown as follows:

First, the IA is conducive to the sustainable development of digital enterprises directly. As a new production factor, digital technology modifies the coefficient of technology in the production function model [27], and the capital-labor substitution rate is likely being increased [28]. The substitution function of information technologies affects the IA’s impact on the sustainable development of enterprises significantly. In addition, the Internet and information technologies optimize the allocation of factors due to the improved supply-and-demand information symmetry, eliminating economic bubbles [18]. However, the impact of IA varies with the innovation and application of information technology. Generally, the higher degree of informatization of enterprises, the stronger elasticity of labor substitution by digital technology [29], and the higher possibility of a positive effect of IA on enterprise efficiency.

Second, the IA facilitates the implementation of PP and mitigates the negative impact of AM on the sustainable development of enterprises. The IA improves the screening ability of the authorities and the accuracy of the selection of beneficiaries, upgrading the traditional monitoring methods [30]. Information, digital and intelligent monitoring technologies strengthen the supervision over the AM and implementation of policy dividends. Therefore, the IA ensures the R&D specialization of policy dividends, optimizes dividends’ allocation efficiency, and positively affects the efficiency of enterprises.

Third, the IA has a negative effect on the effect of PP and the AM’s impact on the sustainable development of enterprises. The innovation and application of digital technology require professional labor skills, adequate digital infrastructures, and smooth networks. The degree of similarity between the current state and the optimal state also affects the performance of IA [31]. Therefore, the following hypothesis is proposed:

Hypothesis 3 (H3): *Under the premise that other conditions remain unchanged*, *the IA positively affects the sustainable development of digital enterprises*.
Hypothesis 4 (H4): *Under the premise that other conditions remain unchanged*, *the IA moderates the effect of AM on the sustainable development of digital enterprises*.

## Methodology, index selection, and data source

### Model setting

#### AM’s influence on the efficiency effect of PP

Instrumental variable methods, difference methods, sensitive analysis, and RDD are the common methods of policy effect testing. The difference methods, which are the most similar to random experiments and considered quasi-natural experiment designs, can be used to overcome the endogenous problem in parameter estimation. This study aimed to investigate the AM’s impact on the sustainable development of enterprises and IA’s moderating effect. The the recognition of high-tech enterprises was regarded as a quasi-natural experiment. PSM-DID, as proposed by Heckman et al. [32], was used to detect the "true" and "fake" digital enterprises. As reported by relevant studies [5, 33, 34], PSM was first used to match the control group and treatment group, and the control group that was most similar to the treatment group was selected to increase the comparability of the samples. Secondly, DID was used to analyze AM’s impact on enterprises’ sustainable development. The following model was set:

$$EFF_{it}=a_{0}+a_{1}HiT_{it}+a_{2}HiT_{it}*POST_{it}+B_{t}+\beta X+\varepsilon_{r}$$ (1)

Where, *EFF* was digital enterprise efficiency, including total factor productivity (*TFP*), scale efficiency (*SE*), technical efficiency (*TE*), and technological progress (*TECH*), representing the sustainability of digital enterprises, *i* was a digital enterprise, *t* was the year, *βt* was the fix-effect virtual variable. Virtual variable *POST* was whether the enterprise was identified as a high-tech enterprise. Virtual variable *HiT* was whether the enterprise had passed the high-tech enterprise identification. *X* was the control variable. a0 was the constant, and a1 and a2 were the coefficients of the corresponding variables. a2 was the impact of Administrative Measures on enterprise efficiency. When a2 was significantly positive, it meant the PP positively affected efficiency. When it was negative, it meant the PP had a negative effect on efficiency.

Based on the model (1), set a model as follows:

$$EFF_{it}=a_{0}+a_{1}HiT+a_{2}HiT_{it}*POST_{it}+a_{3}PsdHiT_{it}+a_{4}PsdHiT_{it}*POST_{it}+B_{t}+\beta X+\varepsilon_{r}$$ (2)

Where *PsdHiT* was AM. a4 was the impact of AM on the sustainable development of digital enterprises on "fake" digital enterprises. The difference of values of a2 in models (1) and (2) was the impact of AM on the sustainable development of "true" digital enterprises. When the value of a2 in model (2) was less than that in model (1), the AM had a negative effect. When the value of a2 in model (1) was higher, the AM had a positive effect.

#### IA’s effect

To verify the direct effect of IA on the sustainable development of digital enterprises, a direct relationship model was set with enterprise efficiency as the dependent variable and enterprise IA (*Inf*) as the independent variable as follows:

$$EFF_{it}=a_{0}+a_{1}Inf_{it}+a_{2}HiT_{it}*POST_{it}+B_{t}+\beta X+\varepsilon_{r}$$ (3)

To verify the moderating effect of IA in the AM’s effect on the sustainable development of digital enterprises, based on model (3), model (4) was set as follows:

$$EFF_{it}=a_{0}+a_{1}Inf_{it}+a_{2}HiT_{it}*POST_{it}+a_{3}Inf_{it}*HiT_{it}*POST_{it}+B_{t}+\beta X+\varepsilon_{r}$$ (4)

Where Inf*HiT*POST was a cross-term of passing high-tech identification (HiT*POST) and IA (*Inf*). From the coefficient a1 of *Inf* in model (3) and the coefficient a3 of Inf*HiT*POST in model (4), we had the moderating effect of IA’s impact on the sustainable development of digital enterprises. If the value of a3 in model (4) was positive, the IA strengthened the effect of model (3), and if the value of a3 in model (4) was negative, the IA weakens the effect of model (3).

To verify the moderating effect of IA in the impact of AM’s effect, based on the idea of the Double Moderating Effect Model, model (5) was set by adding the AM variable (PsdHit*POST) to model (4), and based on model (5), set model (6) by adding the cross terms of AM variable (PsdHit*POST) with IA (*Inf*).

$$EFF_{it}=a_{0}+a_{1}Inf_{it}+a_{2}HiT_{it}*POST_{it}+a_{3}PsdHiT_{it}*POST_{it}+B_{t}+\beta X+\varepsilon_{r}$$ (5)

$$\begin{array}{l} EFF_{it}=a_{0}+a_{1}Inf_{it}+a_{2}HiT_{it}*POST_{it}+a_{3}PsdHiT_{it}*POST_{it}\\ +a_{4}Inf_{it}*HiT_{it}*POST_{it}+a_{5}Inf_{it}*PsdHiT_{it}*POST_{it}+B_{t}+\beta X+\varepsilon_{r} \end{array}$$ (6)

Where from a2 in model (5) and a4 in model (6), we had the moderating effect of the IA’s impact on the sustainable development of "true" digital high-tech information. If the value of a4 in model (6) was positive, the IA strengthened the effect in model (5); if the value of a2 in model (6) was negative, the IA weakened the effect in model (5).

From a3 in model (5) and a5 in model (6), we had the moderating effect of IA’s impact on the sustainable development of "fake" digital enterprises. When a5 in model (6) was positive, the IA strengthened the effect of model (5). When a5 in model (6) was negative, the IA weakened the effect of model (5).

### Variable definition and data source

#### Variable definition

The sustainable development of digital enterprise efficiency (EFF). There are mainly two measurement approaches for the development of DE. One evaluates the DE index by building a comprehensive evaluation index system, including informatization indicators and networking indicators, by adopting the entropy or index method. The other is productivity evaluation by building an evaluation index system, including input and output indicators, employing the DEA and Malmquist index methods [35, 36]. The latter was adopted because the results by empoying it can show the degree of sustainable development. The DEA-Malmquist method was used to measure the relative efficiency of the enterprise [37, 38], using labor and capital investement as inputs and profit and invisibale assets as outputs. The total payroll measured labor input. The total business cost of the enterprise measured by capital investment. The net profit represented the profit output. The net intangible assets represented the intangible assets.

*AM (PsdHiT)*. According to Yang GH, et al. (2017) [5] and the prerequisites for policy dividend granting in the Administrative Measures when the sales revenue was less than 50 million yuan, and the proportion of the enterprise’s R&D investment to the sales revenue was [6%, 7%), the value of *PsdHiT* was 1. When the sales revenue was more than 50 million yuan and less than 200 million yuan, and the proportion is [4%, 5%), the value of *PsdHiT* was 1. When the total revenue was higher than or equal to 200 million yuan, and the proportion was [3%, 4%), the value of *PsdHiT* was 1, and the rest was 0.

Whether the enterprise was recognized as a high-tech enterprise (*HiT*) and the virtual variable of the year after an enterprise was recognized as a high-tech enterprise (*POST*). According to the idea of the PSM-DID method, the value of the variables was decided as follows: When the enterprise was identified as a high-tech enterprise during the inspection, the *HiT* value was 1, or it was 0. After the enterprise was recognized as a high-tech enterprise, the value of *POST* was 1, or it was 0.

*IA (Inf)*. IA refers to the information interaction ability or connectivity between individuals or regions [39]. With Internet technology, information is transmitted using digital coding [40], so the IA level is proportional to the level of its digitization level. Therefore, according to Yuan C. et al. (2021), Wu F. et al., (2021) and He F. et al. (2019) [41–43], the frequency of digital keywords was evaluated by adopting the MDA analysis of annual reports. A total of 184 terms like "AI technology", "block chain technology", "cloud computing tech", "big data technology", and "digital technology application" etc. were used as keywords. Then, the logarithm was taken as the value of IA. The frequency of keywords divided by the total number of words in the annual report was used as a surrogate variable for IA to ensure the robustness of the results.

*Control variables*. It included the equity ratio (*ownershipp*), asset-liability ratio (*balance*), equity concentration (*con*), whether the chairman and general manager are the same person (*chairman*), and financial leverage (*lever*). The equity ratio was measured by the total liabilities divided by shareholders’ equity. The asset-liability ratio measures the ratio of total liabilities to total assets. Whether the chairman and general manager are the same person was 1 if the answer was yes or it was 0. The ratio of change rate of earnings per share of common stock to change rate of EBIT measured the financial leverage.

#### Data sources

Descriptive statistics for variables and data are shown in Table 1. The samples were selected from the enterprise list of the DE sector of the Shenzhen and Shanghai Stock Exchanges, excluding those listed or delisted in or after 2007, ST enterpreses, and ST* enterprises. The data of 1200 digital enterprises between 2007 to 2021 was from the Guotai’an database (www.gtarsc.com/) and the annual reports of listed companies in the Shenzhen and Shanghai Stock Exchanges. Based on normalized data, evaluation was done by using Stata 15.0.

**Table 1:** Descriptive statistics of the data.

		Variable	Symbol	Sample Number	Mean	Standard Error	Min Value	Max Value
**The main dependent variable**		Total factor productivity	*TFP*	18,000	1.009	0.0417	0.779	1.483
		Technical efficiency	*TE*	18,000	1.001	0.0330	0.826	1.236
		Scale efficiency	*SE*	18,000	1.000	0.0156	0.882	1.278
		Technological progress	*TECH*	18,000	1.009	0.0363	0.835	1.342
**Efficiency measurement index system**	**Input**	Labor input	lnl	14,453	17.010	1.747	6.658	23.48
		Capital input	lnk	16,933	18.890	7.706	0.000	28.020
	**Output**	Profit	lny	13,180	18.680	1.470	11.440	24.600
		Intangible assets	lni	14,379	18.270	1.811	0.000	24.030
**The main independent variables**		Company research and development exercise, vertical virtual variable	*PsdHiT*	14,184	0.241	0.428	0.000	1
		Whether companies are identified as high	*HiT*	14,184	0.514	0.500	0.000	1
		Virtual variable of the year after obtaining the high enterprise recognition	*POST*	18,000	0.723	0.447	0.000	1
		IA	*Inf*	14,142	9.589	20.110	0.000	264.000
**control variable**		Equity ratio	*ownershipp*	16,375	1.100	6.282	-104.800	685.900
		Asset-liability ratio	*balance*	16,375	0.432	1.010	0.000	96.960
		Equity concentration	*con*	16,375	28.800	17.900	0.000	89.990
		Whether the chairman and general manager are the same person	*chairman*	16,375	0.269	0.444	0.000	1
		Financial leverage	*lever*	16,375	1.238	3.128	-81.340	271.000

## Results and analysis

### The AM’s impact on the sustainable development of digital enterprise

Two-way fixed-effect Muti-stage DID regression and PSM-Muti-stage DID model results are shown in Table 2. The results of both regressions were similar, indicating that the PSM-DID method was feasible.

**Table 2:** OLS regression results and PSM-DID regression results for model (1).

	Muti-stage DID model regression				PSM-Muti-stage-DID regression results			
	ln*TFP*	ln*TE*	ln*TECH*	ln*SE*	ln*TFP*	ln*TE*	ln*TECH*	ln*SE*
HiT *POST	0.0013** (-0.001)	0.0010** (-0.001)	0.0009** (-0.001)	-0.0006*** (-0.000)	0.0013** (-0.001)	0.0011** (-0.001)	0.0009** (-0.001)	-0.0006*** (-0.000)
**i.time**	Yes	Yes	Yes	Yes	Yes	Yes	Yes	Yes
**i.id**	Yes	Yes	Yes	Yes	Yes	Yes	Yes	Yes
***X***	Yes	Yes	Yes	Yes	Yes	Yes	Yes	Yes
**_cons**	2.99E-05 (0.000)	5.73E-05*** (0.000)	-4.54E-05** (-0.000)	1.79E-05 (0.000)	2.91E-05 (0.000)	5.82E-05*** (0.000)	-4.70E-05*** (0.000)	1.79E-05 (0.000)
**N**	14184	14184	14184	14184	14176	14176	14176	14176
**adj.R-sq**	0.6701	0.7356	0.8072	0.6700	0.6703	0.7361	0.8072	0.6701

Note: Standard error is included in parentheses.

*, ** and *** are significant at the levels of 10%, 5% and 1% respectively.

Table 3 shows the two-way fixed-effect PSM-DID regression results of model (2). The results showed that: (1) The coefficient between ln*TFP* and *HiT* was of the same direction as that of model (1), indicating that passing the recognition of high-tech enterprises is conducive to the optimization of efficiency, and the positive effect was significant because of the optimization of technological progress and scale efficiency. This confirmed hypothesis 1.

**Table 3:** The PSM-DID regression results of model (2).

	ln*TFP*	ln*TE*	ln*TECH*	ln*SE*
HiT*POST	0.0033*** (-0.001)	0.0008 (-0.001)	0.0024*** (-0.001)	0.0001 (0.000)
***PsdHiT***	0.0032*** (-0.001)	0.0006 (-0.001)	0.0015** (-0.001)	0.0010*** (0.000)
PsdHiT*POST	-0.0050*** (-0.001)	0.0006 (-0.001)	-0.0037*** (-0.001)	-0.0019*** (-0.001)
**control variables**	yes	yes	yes	yes
**i.time**	Yes	Yes	Yes	Yes
**i.id**	Yes	Yes	Yes	Yes
***X***	Yes	Yes	Yes	Yes
**_cons**	2.93E-05 (0.000)	5.79E-05*** (0.000)	-4.62E-05*** (0.000)	1.76E-05 (0.000)
**N**	14184	14184	14184	14184
**adj.**	0.6706	0.7362	0.8075	0.6704

Note: Standard error is included in parentheses.

*, ** and *** are significant at the levels of 10%, 5% and 1% respectively.

(2) The coefficient between ln*TFP* and *PsdHiT* was -0.0050, indicating that AM hindered the sustainable development of "true" high-tech enterprises, which confirmed hypothesis 2 of this paper. The negative effect of AM was mainly because of the negative effect on the SE and TECH of "true" digital enterprise.

### The direct efficiency effect of IA on enterprise

Table 4 shows the results of a direct efficiency effect of IA on enterprise (model 3). The coefficient between ln*TFP* and *Inf* was 1.47E-04, and it was significant. It confirmed hypothesis 3. The positive effect of IA on the efficiency of digital enterprises was from the effect on scale efficiency (the coefficient was 3.19E-05) and technical efficiency (the coefficient was 1.21E-04). In contrast, the negative effect on TE was not significant.

**Table 4:** The direct effect of IA on enterprise efficiency.

	(1)	(3)	(5)	(7)
	ln*TFP*	ln*TE*	ln*TECH*	ln*SE*
***Inf***	1.47E-04*** (0.001)	-6.15E-06 (0.001)	1.21E-04*** (0.001)	3.19E-05*** (0.001)
HiT*POST	0.0012* (-0.001)	8.81E-04* (-0.001)	9.76E-04** (-0.001)	-6.54E-04*** (-0.001)
**control variables**	yes	yes	yes	yes
**i.time**	Yes	Yes	Yes	Yes
**i.id**	Yes	Yes	Yes	Yes
**_cons**	3.55E-05 (0.001)	5.53E-05*** (0.001	-3.86E-05** (0.001)	1.88E-05 (0.001)
**N**	14121	14121	14121	14121
**adj.R-sq**	0.6738	0.7372	0.8098	0.6716

Note: Standard error is included in parentheses.

*, ** and *** are significant at the levels of 10%, 5% and 1% respectively.

### The moderating effect of IA

Table 5 shows the results of model (4). The coefficient between HiT*Inf and ln*TFP* was negative. The coeefficent between HiT*Inf and ln*TE* was significantly negative, but the coefficients between HiT*Inf and ln*TECH* and between HiT*Inf and ln*SE* were positive, indicating the IA deteriorated the positive effect of the high-tech enterprises recognition on the TFP of digital enterprises, i.e., IA had a negative moderating effect. The negative moderating effect was primarily from the negative impact on the technological efficiency of digital enterprises. In contrast, the positive intermediate effect on the scale efficiency and technology progress was not significant or sufficient.

**Table 5:** Results of the indirect effect of IA on the efficiency of "True" digital enterprises.

	(1)	(2)	(3)	(4)
	ln*TFP*	ln*TE*	ln*TECH*	ln*SE*
***Inf***	1.49E-04*** (0.000)	1.30E-05 (0.000)	1.05E-04*** (0.000)	3.10E-05*** (0.000)
HiT*POST	0.0012* (-0.001)	0.0012*** (-0.001)	0.0007 (-0.001)	-0.0007*** (0.000)
HiT *POST*Inf	-3.84E-06 (0.000)	-3.18E-05* (0.000)	2.67E-05 (0.000)	1.52E-06 (0.000)
**control variable**	Yes	Yes	Yes	Yes
**i.time**	Yes	Yes	Yes	Yes
**i.id**	Yes	Yes	Yes	Yes
**_cons**	0.0080*** (-0.001)	-0.0006 (-0.001)	0.0097*** (-0.001)	-0.0011*** (0.000)
**N**	14121	14121	14121	14121
**adj.R-sq**	0.6738	0.7372	0.8098	0.6716

Note: Standard error is included in parentheses.

*, * * and * * * are significant at the levels of 10%, 5% and 1% respectively.

Table 6 shows the results of models (5) and (6), indicating the moderating effect of IA on the sustainable development of "fake" digital enterprises. Comparing the results of (1) with (5), (2) with (6), (3) with (7), and (4) with (8), the coefficient between PsdHiT*Inf and ln*TFP* was significantly negative, indicating that, as for the "fake" digital enterprises, the IA had a negative moderating effect on the positive effect on TFP, indicating that it weakened the positive effect on the efficiency of "fake" digital enterprises. It confirmed hypothesis 4, indicating that IA moderated the AM’s impact on the sustainable development of digital enterprises. The negative moderating effect in the TE of digital enterprises was significant, while the others were not. The coefficients between PsdHiT*Inf and ln*TECH* and between PsdHiT*Inf and ln*SE* were significantly negative, implying that the negative intermediate effect of IA on the efficiency effect was due to the negative intermediate effects on the technology progress and scale efficiency.

**Table 6:** The moderating effect of IA on the sustainable development of "True" and "Fake" digital enterprises.

	(1)	(2)	(3)	(4)	(5)	(6)	(7)	(8)
	ln*TFP*	ln*TE*	ln*TECH*	ln*SE*	ln*TFP*	ln*TE*	ln*TECH*	ln*SE*
***Inf***	1.45E-04*** (0.000)	-6.20E-06 (0.000)	1.19E-04*** (0.000)	3.18E-05*** (0.000)	2.72E-05 (0.000)	-7.93E-06 (0.000)	2.22E-05** (0.000)	1.32E-05** (0.000)
HiT*POST	0.0023*** (-0.001)	5.05E-04 (-0.001)	0.0020*** (-0.001)	-1.88E-04 (0.000)	0.0024*** (-0.001)	7.76E-04 (-0.001)	0.0018*** (-0.001)	-1.31E-04 (0.000)
*HiT* *POST *Inf					-1.44E-05 (0.000)	-2.83E-05 (0.000)	1.96E-05 (0.000)	-5.60E-06 (0.000)
***PsdHiT***	-0.0026*** (-0.001)	9.32E-04 (-0.001)	-0.0024*** (-0.001)	-0.0012*** (0.000)	-0.0033*** (-0.001)	0.0011 (-0.001)	-0.0029*** (-0.001)	-0.0015*** (0.000)
PsdHiT*POST*Inf					2.72E-05 (0.000)	-7.93E-06 (0.000)	2.22E-05** (0.000)	1.32E-05** (0.000)
**control variable**	yes	yes	yes	yes	yes	yes	yes	yes
**_cons**	0.0082*** (-0.001)	-4.98E-04 (-0.001)	0.0097*** (-0.001)	-0.0010*** (0.000)	0.4430 (-0.333)	0.3940*** (-0.15)	-3.7170*** (-0.413)	2.6520*** (-0.341)
**N**	14117	14117	14117	14117	14117	14117	14117	14117
adj.R-sq	0.6739	0.7372	0.8099	0.6718	0.6739	0.7372	0.81	0.6719

Note: Standard error is included in parentheses.

*, * * and * * * are significant at the levels of 10%, 5% and 1% respectively.

## Related tests

### Robust test and co-integration inspection

Methods including LLC, IPS, ADF Fisher, PP Fisher, Breitung, Hadri, and HT tests were used for the robust test. The p values showed that all variables did not support the original hypothesis of containing unit root and met the requirements of robustness. Panel Rho statistic, panel PP statistic, panel ADF statistic, group PP statistic, and group ADF statistic were also used. The variables passed the co-integration test according to the outcomes of the F, LR, and Hausman test.

#### Test of the effect of PSM matching

To test the matching effect of the PSM method, we compared the density function diagrams before and after the PSM method matching, and found that the density function of the matched enterprises in the control group was comparable to that in the experimental group. We also compared the differences in enterprise characteristics between "true" and "fake" digital high-tech enterprises before and after the PSM method matching. It was found that there was no significant difference in enterprise characteristics between the two groups of samples after matching. In light of this, we concluded that the experimental and control groups matched by the PSM methods were highly comparable.

### DID parallel trend hypothesis test

The validity of the evaluation results of the DID model depends on whether the "parallel trend hypothesis" can be satisfied. The results of the parallel trend hypothesis test of the experimental and control groups’ samples showed that the only difference was that the experimental group passed the high-level identification while the control group did not. In addition, we used the dynamic regression model to test the impact of high-tech enterprise identification on enterprise efficiency. From the parallel trend diagram of DID model, it showed that the difference between TFP and its efficiency decomposition indexes SE, TE, and TECH of digital enterprises was narrowed after they were recognized as high-tech enterprises, showing that the DID model met the parallel trend assumption.

### Placebo test

To rule out the possibility that the efficiency changes of enterprises after obtaining high-tech enterprise recognition were due to time trends, we conducted a placebo test by randomly selecting 100 digital enterprises and their recognition years. The PSM method matched these samples, thus obtaining the PSM sample results. The results showed that the empirical results were consistent with those of the PSM samples mentioned above, confirming that the time trend did not cause the PSM-DID regression results.

## Discussion

The IA enables better accountability in public bureaucracies through E-governance initiatives, and understanding the IA has become essential to describing China’s economic and social development in the new era [44]. For a long time, the AM issue is the key to addressing sustainable development.

Firstly, it is clear from the analysis of this study that it is passing the recognition of high-tech enterprises while the AM hindered the sustainable development of digital enterprises. As for the "true" high-tech digital enterprises, the AM improved the scale efficiency, which could be because the "fake" and the "true" digital enterprises were collaborative. The growing "fake" digital enterprises supplemented, strengthened and extended the industrial chain and upgraded the industrial structure [26], forming a feedback effect on the sustainable development of "true" digital enterprises.

However, for the "fake" high-tech enterprise, being a high-tech enterprise is not conducive to optimizing their efficiency, particularly their scale efficiency or technological progress. Although the AM had a negative effect on the allocation of policy resources, the policy dividends were conducive to the expanding market scale and improving the market environment, which was also conducive to the optimizing "true" digital enterprises. However, the AM cost might have a siphon effect on the operating and R&D funds, causing a negative effect on "fake" enterprises.

Secondly, the IA was conducive to the sustainable development of digital enterprises. As enterprises have reached a certain level of informization, digitization and networking, the application of information technology in digital enterprises positively impacts the management of R&D and production planning. In addition, it was conducive to breaking the information bottleneck to the demand side and facilitate scale expansion, thereby optimizing scale efficiency.

Thirdly, the IA’s moderating effect on high-tech enterprises was insufficient. Although IA should, theoretically, have a positive effect, which was conducive to alleviating the negative effect of AM on the sustainable development of digital enterprises, the positive impact of IA on output efficiency varied with the development of DE [38]. The DE was still developing then, and the integration of inform, intelligence, and digital technology with the real economy was low. Therefore, the effect of IA was not yet extinguished.

Finally, the IA’s moderating effect on "fake" high-tech enterprises was significantly negative, indicating that IA strengthened the negative effect in "fake" enterprises, especially on the effect of the technological progress and the scale efficiency.

## Conclusions

A theoretical mechanism was analyzed from the micro perspective of the enterprise to determine the impact of IA and AM on the sustainable development of digital enterprises. China’s 1200 listed digital enterprises efficiency between 2007 and 2021 was estimated based on the DEA-Malmquist index model. The IA value was determined based on the big data crawler method. Based on the Administrative Measures, empirical analyses were conducted using models such as the panel PSM-DID method and the double moderating effect model. The following conclusions were obtained:

First, the AM affected the sustainable development of digital enterprises heterogeneously. As for the "true" high-tech digital enterprises, the AM improved the scale efficiency, which could be of the feed back effect of AM from the "fake" ones to the the whole industry. The AM hindered the sustainable development of "fake" digital enterprises mainly because of the negative effect on their technology progress and scale efficiency.

Second, the IA had a positive direct effect on the sustainable development of digital enterprises due to its effect on their technological efficiency and scale efficiency.

Third, the IA’s moderating effect on the AM’s effect on the sustainable development of digital enterprises was heterogeneous. IA generally did not improve the efficiency effect on "true" enterprises but significantly strengthened the negative effect of AM on the efficiency of "fake" digital enterprises, especially on the technology progress and scale efficiency.

We draw the following enlightenment: First, in addition to encouraging more R&D investments, we should strengthen and accelerate the construction of a "smart government", thereby strengthening the identification and supervision ability of the authorities for the positive effect of IA. Second, accelerate the cultivation and introduction of talents, optimize the human resource structure, release a positive effect on the TECH of digital enterprises, and further promote the TECH of enterprises. Third, cultivate the digital product and service market, break the supply-and-demand information bottleneck with the aid of digital technology, and direct enterprises to increase their market scale, thereby enhancing the SE and TFP of digital enterprises.

The main contributions of this research could be: first, this research focused on the opportunities brought by the digital economy because it improves information asymmetry and limits accounting manipulation behavior. Second, this research studied the AM’s impact on "true" and "fake" digital enterprises’ and the IA’s direct and moderating effects rather than taking all the digital enterprises as a whole object, deepening the study and proposing targeted suggestions.

This study has limitations: first, due to the lack of official statistics on the digital economy, this study can only be based on the data of digital enterprises. Second, it fails to account for the more direct evidence of the feedback effect of the "fake" enterprises on the "true" ones. If it can be investigated, it will aid in overcoming the negative impact of accounting manipulation on the industrial efficiency effect of preferential policies and promote the sustainable growth of the digital industry.
